# Determinants of self-reported chronic disease diagnoses among older persons in South Africa

**DOI:** 10.4102/phcfm.v16i1.4425

**Published:** 2024-04-30

**Authors:** Maatla D. Temane, Stephina K. Mbele, Mluleki Tsawe

**Affiliations:** 1 Research Unit, Centre for Statistical Analysis and Research, Johannesburg, South Africa; 2 Department of Population Studies and Demography, Faculty of Humanities, North-West University, Mafikeng, South Africa; 3 Population and Health Research Focus Area, Faculty of Humanities, North-West University, Mafikeng, South Africa

**Keywords:** older persons, cancer, diabetes, hypertension, arthritis, stroke, prevalence, disability

## Abstract

**Background:**

Chronic diseases tend to affect the quality of life for older persons worldwide, especially in resource-constrained developing countries. Chronic diseases contribute to a large number of deaths among the population of South Africa.

**Aim:**

This study examines the determinants of self-reported chronic disease diagnoses among older persons in South Africa.

**Setting:**

The study setting was South Africa.

**Methods:**

Cross-sectional data from the 2019 South Africa General Household Survey were analysed (*n* [weighted] = 4 887 334). We fitted a binary logistic regression model to determine the relationship between socio-demographic factors and being diagnosed with self-reported chronic diseases.

**Results:**

We found that at least 5 in 10 older persons were diagnosed with self-reported chronic disease. The bivariate findings showed that age, population group, sex, marital status, level of education, disability status, household composition and province were significantly associated with self-reported chronic disease diagnoses. At the multivariate level, we found that age, sex, population group, marital status, educational level, disability status, household wealth status, household composition and province were key predictors of self-reported chronic disease diagnoses.

**Conclusion:**

We found that various factors were key determinants of being diagnosed with self-reported chronic diseases. This study offers important insights into the main correlations between older adults and self-reported chronic illness diagnoses. More study is required on the health of the elderly as it will help direct policy discussions and improve the development of health policies about the elderly.

**Contribution:**

This study highlights the need for a better understanding of, and continued research into, the determinants health among older populations to guide future healthcare strategies.

## Introduction

The well-being of elderly individuals, concerning non-communicable diseases (NCDs), is a global public health issue. Non-communicable diseases, also referred to as chronic diseases, arise from a complex interplay of genetic, physiological, environmental and behavioural factors, leading to prolonged durations of illness. They encompass a broad range of conditions such as cardiovascular diseases, cancer, chronic respiratory diseases and diabetes, among others.^1^ Non-communicable diseases and related health concerns have decreased the quality of life for older adults. The complex and extensive healthcare requirements of older individuals with NCDs will pose significant challenges to healthcare systems in low- and middle-income countries.^2^ Over the past three decades, NCDs have been progressively identified as a major cause of disability and death. Research shows that NCD deaths increased from over 7 million in 1990 to over 51 million in 2010.^3^ Chronic NCDs accounted for over 80% of deaths among older adults in 2000, with cardiovascular disorders being the leading cause of death in South Africa. Heart disease and stroke were responsible for approximately a third of deaths in South Africa.^4^ The expanding population of older adults and the growing prevalence of chronic illnesses raise concerns regarding successful ageing and the adequacy of healthcare services for this demographic.^5^

Various studies, looking at chronic diseases among older persons, have been conducted in South Africa.^6,7,8,9,10^ These studies focussed on various aspects of adult chronic health such as the relationship between multimorbidity and disability,^11^ financial condition (which may be defined as one’s finances) and its relationship with chronic diseases,^6,12^ as well as NCDs and multimorbidity.^7,9^ Most of these studies have centred on persons aged 50 years and older.^6,8,9^ Although much of the research has focussed on older persons’ health, little research has specifically focussed on health issues among older persons. This study focusses on older persons whom we define as persons aged 60 years and older. In South Africa, the *Older Persons Act* 13 of 2006 defines older persons as those who are at least 60 years or older.^13^

Several factors have been established as determinants of chronic diseases among older persons. This study highlights the importance of age and sex as determinants of chronic diseases. Age has been noted as an important determinant of chronic diseases. Poor health outcomes and chronic diseases tend to increase with age.^14,15,16^ In a rural Vietnam study, it was discovered that individuals in their seventies were more prone to chronic diseases than those in their early sixties.^17^ Other studies have found an association between sex and having chronic diseases.^18,19,20,21^ A study by,^17^ revealed that females are more likely to have chronic diseases as compared to males. A study^21^ found that medical conditions differ according to sex, for instance, the prevalence of depression was found to be higher among females than males and one-third of females were reported to have lived with chronic conditions than males.

We used the Commission on Social Determinants of Health (CSDH) framework to determine how various sociodemographic factors, such as age, sex, education, race and household wealth, among others, influence older people’s health outcomes.^22^ This framework argues that various factors influence health beyond biological factors.^23,24^ This framework assists in presenting important information that can be used by policymakers, researchers and governments to assist in reducing inequities and promoting better health outcomes.^25^ With this, the main objective of the study was to examine the determinants of self-reported chronic disease diagnoses among older persons in South Africa. We also aimed to examine the prevalence of self-reported chronic disease diagnoses among elderly persons in South Africa and to investigate the socio-demographic factors associated with self-reported chronic disease diagnoses.

## Research methods and design

### Study design

The study followed the cross-sectional study design. Cross-sectional secondary data were used from the 2019 General Household Survey (GHS) and focussed on persons aged 60 years and older.

### Setting

The setting of the study is South Africa, which comprising 9 provinces, each further subdivided into 52 districts, including 8 metropolitan areas and 226 local municipalities. As of 2022, the total population of South Africa stood at 62 million individuals.^26^ Individuals classified as older persons, aged 60 and above, make up over 8.5% of the total population.^26^ The Eastern Cape province had the highest number of older persons at 11.6%, followed by Western Cape at 10.7%, and the Northern Cape with the lowest at 10.1%.^26^

### Data source

The 2019 GHS, which is secondary cross-sectional data, was utilised, encompassing a weighted sample of 4 887 334 individuals aged 60 years and above. The decision to employ the 2019 GHS stemmed from concerns over the data integrity of more recent GHS iterations, which may have been impacted by the repercussions of the coronavirus disease 2019 (COVID-19) pandemic. The GHS is a household-based cross-sectional survey, representative at the national level, covering all nine provinces within the country. The target population for this survey comprised all private households across the nation.^27^

### Study population, sampling strategy, inclusion and exclusion criteria

**FIGURE 1:**
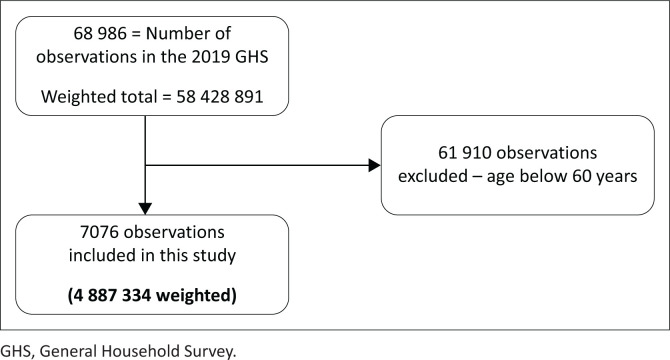
Flow diagram of the study participants and study sample.

The survey excluded the population living in institutionalised settings ‘such as students’ hostels, old-age homes, hospitals, prisons, military barracks’ and others. The survey used a two-stage stratified sampling design.^27^ In the first stage, primary sampling units (PSUs) were selected, and in the second stage, dwelling units (DUs) were selected.^27^ Primary sampling unit is a geographical or administrative unit used for sampling; it helps ensure geographical representation in the sample.^28,29^ Whereas a DU is a physical unit where people reside and represent the actual places where data are collected.^27^ The PSUs are selected first in the sampling process and thereafter DUs are selected within those PSUs.^28,29^ The response rate of the 2019 GHS was 87.2%.^27^ Further information about the GHS sampling, study setting and weighting can be found in the 2019 GHS metadata report.^27^ Figure 1 explains the steps taken to reach our study sample.

### Study variables

The data were extracted from GHS 2019. The study’s dependent variable is based on individuals’ self-reported chronic disease diagnoses, derived from their self-reported health status. The participants were asked about their chronic disease diagnoses as reported by a physician: ‘*Has a doctor/nurse/other healthcare worker at a clinic/hospital/private practice ever told (name) that he/she has/had any of the following*?’^27^ In the survey, conditions such as: (1) asthma, (2) diabetes, (3) cancer, (4) HIV (human immunodeficiency virus) and AIDS (acquired immunodeficiency syndrome), (5) hypertension or high blood pressure, (6) arthritis, (7) stroke, (8) heart attack or myocardial infarction, (9) tuberculosis, (10) mental illness, (11) epileptic seizure, (12) meningitis and sinusitis, (13) pneumonia, (14) bronchitis, (15) high cholesterol, (16) osteoporosis and (17) malaria were included.^27^ The survey asked respondents to respond with a ‘yes’ or ‘no’ to each item in the list. This study focusses only on the following diagnosed diseases: diabetes, hypertension, cancer, arthritis and stroke.

#### Explanatory variables

We included 10 explanatory variables in this study. Table 1 describes the selected explanatory variables.

**TABLE 1:** Description of the selected explanatory variables.

Variable	Definition	Code
Age group	This is the respondent’s age in completed years (grouped into 5-year age groups).	1 = 60–64
		2 = 65–69
		3 = 70–74
		4 = 75–79
		5 = 80 +
Sex	This is the respondent’s reported sex.	1 = Male
		2 = Female
Population group	This is the respondent’s population group based on South Africa’s classifications.	1 = Black people
		2 = Coloured people
		3 = Indian people and Asian people
		4 = White people
Marital status	This is the respondent’s reported marital status.	1 = Married
		2 = Cohabiting
		3 = Never married
		4 = No longer married
Educational level	This is the respondent’s reported highest level of education.	1 = No schooling
		2 = Primary
		3 = Secondary
		4 = Higher
		5 = Other
Disability status	This is the respondent’s reported disability status (i.e., functional limitation). For more information about the disability questions see the Washington Group on Disability Statistics website (see https://www.washingtongroup-disability.com/).	1 = No difficulty
		2 = Some difficulty
		3 = A lot of difficulty
		4 = Cannot do at all
Household wealth status	This is the respondent’s wealth status derived based on the PCA using household assets; similar to the methods used by the DHS (see https://dhsprogram.com/topics/wealth-index/Wealth-Index-Construction.cfm).	1 = Poor
		2 = Average
		3 = Richer
Household composition	This is the respondent’s type of living arrangement. The household composition is derived in a similar manner as used by the GHS. For more information about this variable, see the GHS report (see https://www.statssa.gov.za/publications/P0318/P03182019.pdf).	1 = Lone male
		2 = Lone female
		3 = Nuclear, male-headed
		4 = Nuclear, female-headed
		5 = Extended, male-headed
		6 = Extended, female-headed
		7 = Complex
Geography type	This is the respondent’s geographical location at the time of the survey.	1 = Urban
		2 = Traditional
		3 = Farms
Province	This is the respondent’s province of residence at the time of the survey.	1 = Western Cape
		2 = Eastern Cape
		3 = Northern Cape
		4 = Free State
		5 = Kwa-Zulu Natal
		6 = North West
		7 = Gauteng
		8 = Mpumalanga
		9 = Limpopo

GHS, General Household Survey; DHS, Demographic and Health Survey; PCA, Principal Components Analysis.

### Data analysis

We used Stata version 16 (StataCorp LLC, Texas, USA) to analyse the data in this study.^30^ For this study, three types of analyses were used: univariate, bivariate and multivariate analysis. The univariate analysis includes descriptive statistics. For bivariate analysis, the chi-square test was utilised to show the correlation among diagnoses of chronic disease and selected independent variables. In the multivariate analysis, binary logistic regression was used to analyse the relationship between the selected background characteristics and being diagnosed with a chronic disease. We used the ‘*svy*’ command in Stata to adjust for the complex sampling structure of the data in the analyses. We further used the variance inflation factor (VIF) to test for multicollinearity in the explanatory factors. The multicollinearity test found no collinearity between the variables; the minimum VIF was 1.06, the maximum VIF was 2.05 and the mean VIF was 1.39.

### Ethical consideration

We used secondary data from the 2019 GHS. The data collected by Statistics South Africa followed all the necessary ethical considerations. The collection of data by Statistics South Africa is guided by the fundamental principles of statistics (see more details at https://www.statssa.gov.za/?page_id=361).

## Results

### Sample description

The characteristics of the study sample are presented in Table 2. Based on the findings, older people in the age group 60–64 years were dominating, whereas those in the age group 75+ were less dominating. There were more females than males in the sample. The black population group constituted the largest sample in the study; there were fewer older persons from the Indian and Asian population group. In terms of marital status, the majority of the sample was married, while a few were cohabiting. Those with secondary education made up the largest percentage. The majority of the study sample had no disability difficulty. Over 57% of the study sample was from rich households. Over 29% of the study sample was from extended, female-headed households. Over 64% of the study sample was from urban areas, while 3% was from farm areas. The majority of the study sample was from Gauteng province.

**TABLE 2:** Prevalence of self-reported chronic disease diagnoses by background characteristics.

Characteristics	Prevalence	*N*	%	χ^2^	
				value	*P*
**Age group**				77.5	0.000
60–64	45.5	1 688 426	34.5	-	-
65–69	48.0	1 275 770	26.1	-	-
70–74	57.6	865 397	17.7	-	-
75–79	52.7	528 121	10.8	-	-
80 +	55.5	529 620	10.8	-	-
**Sex**				146.6	0.000
Male	41.8	1 964 263	40.2	-	-
Female	55.7	2 923 071	59.8	-	-
**Population group**				72.5	0.000
Black people	54.9	3 115 365	63.7	-	-
Coloured people	44.0	491 385	10.1	-	-
Indian people and Asian people	43.2	189 309	3.9	-	-
White people	40.5	1 091 275	22.3	-	-
**Marital status**				75.4	0.000
Married	45.7	2 206 526	45.1	-	-
Cohabiting	38.6	148 726	3.0	-	-
Never married	49.0	549 359	11.2	-	-
No longer married	5.9	1 982 722	40.6	-	-
**Educational level**				24.9	0.000
No education	51.5	784 115	16.0	-	-
Primary	54.4	1 447 427	29.6	-	-
Secondary	49.3	1 932 442	39.5	-	-
Higher	41.1	590 376	12.1	-	-
Other	49.3	132 974	2.7	-	-
**Disability status**				98.9	0.000
No difficulty	45.7	2 884 304	59.0	-	-
Some difficulty	56.7	1 261 008	25.8	-	-
A lot of difficulty	57.0	592 843	12.1	-	-
Cannot do at all	53.4	149 180	3.1	-	-
**Household wealth status**				0.5	0.785
Poor	51.4	1 264 943	25.9	-	-
Average	52.4	809 555	16.6	-	-
Rich	49.0	2 812 836	57.6	-	-
**Household composition**				85.3	0.000
Lone male	43.2	222 170	4.5	-	-
Lone female	48.0	257 123	5.3	-	-
Nuclear, male-headed	46.7	1 174 293	24.0	-	-
Nuclear, female-headed	48.9	325 401	6.7	-	-
Extended, male-headed	46.2	1 341 888	27.5	-	-
Extended, female-headed	58.8	1 448 727	29.6	-	-
Complex	45.0	117 732	2.4	-	-
**Geography type**				0.4	0.809
Urban	49.2	3 170 489	64.9	-	-
Traditional	52.0	1 548 795	31.7	-	-
Farms	50.8	168 050	3.4	-	-
**Province**				14.8	0.000
Western Cape	43.7	634 944	13.0	-	-
Eastern Cape	55.8	688 845	14.1	-	-
Northern Cape	49.6	124 647	2.6	-	-
Free State	51.9	265 665	5.4	-	-
KwaZulu-Natal	57.3	851 096	17.4	-	-
North West	52.1	359 075	7.3	-	-
Gauteng	48.6	1 183 106	24.2	-	-
Mpumalanga	52.4	329 609	6.7	-	-
Limpopo	37.1	450 348	9.2	-	-
South Africa	50.2	4 887 334	100.0	-	-

Note: χ^2^, Chi-square tests.

### Prevalence of self-reported chronic disease diagnoses

The results presented in Table 2 indicate the frequency of chronic disease diagnoses as reported by individuals, categorised according to various demographic factors. The results revealed that age, population group, sex, marital status, level of education, disability status, household composition and province were associated with self-reported chronic disease diagnoses. Those aged 70–74 years had a higher prevalence (57.6%) of self-reported chronic disease diagnoses. Females had a higher prevalence (55.7%) of self-reported chronic disease diagnoses. The black population group had a higher prevalence (54.9%) of self-reported chronic disease diagnoses, while it was lower (40.5%) among the white population group. Those who were never married had a higher prevalence (49.0%) of self-reported chronic disease diagnoses, while it was low (5.9%) for those who were no longer married.

**FIGURE 2:**
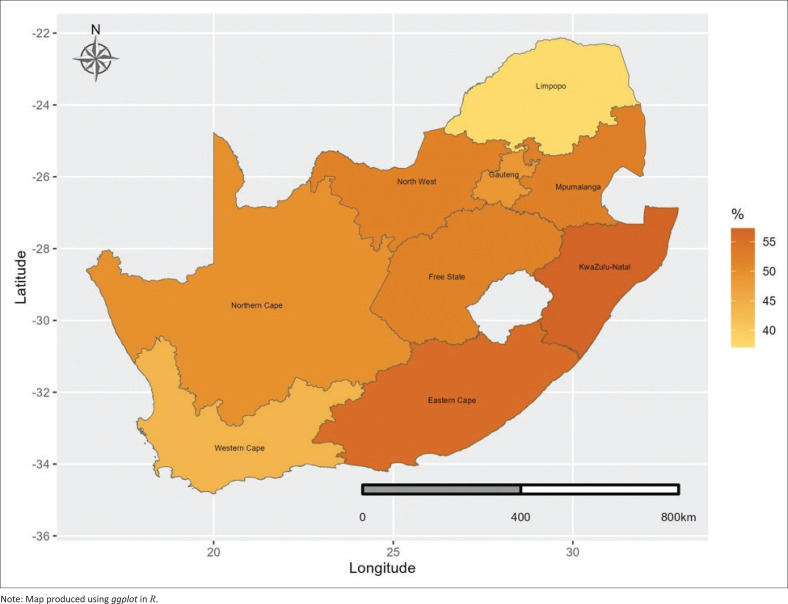
Prevalence of self-reported chronic disease diagnoses by province.

The prevalence of self-reported chronic disease diagnoses was lower for those with higher socioeconomic status. The findings showed that those with primary education had a higher prevalence (54.4%) of self-reported chronic disease diagnoses, while it was lower (41.1%) for those with higher education. In terms of household wealth status, those from average-wealth households had a higher prevalence (52.4%) of self-reported chronic disease diagnoses, while it was lower (49.0%) for those who were from rich households. Those who had a lot of difficulty, in terms of disability status, had a higher prevalence (57.0%) of self-reported chronic disease diagnoses. In terms of household composition, those who were from extended, female-headed households, had a higher prevalence (58.8%) of self-reported chronic disease diagnoses. There were geographical variations in chronic disease diagnoses. The findings showed that those who were from traditional areas had a higher prevalence (52.0%) of self-reported chronic disease diagnoses. In terms of the province, those who were from KwaZulu-Natal (57.3%) and Eastern Cape (55.8%) had a higher prevalence (57.0%) of self-reported chronic disease diagnoses, while it was lower (37.1%) among those from Limpopo (see the visual presentation by province in Figure 2).

### Determinants of being diagnosed with chronic conditions

Table 3 presents the results of the multivariate analysis exploring the factors influencing self-reported chronic disease diagnoses among South Africa’s older population. The analysis revealed several significant findings. Individuals aged 70–74 years were 1.65 times more likely to report chronic diseases (95% confidence interval [CI]: 1.39–1.97) compared to those aged 60–64 years. Similarly, individuals aged 75–79 years had a 1.37 times higher likelihood (95% CI: 1.11–1.70) of reporting chronic diseases compared to the reference group (60–64 years old). Those aged 80 and above were 1.40 times more likely (95% CI: 1.12–1.75) to report chronic diseases compared to the 60–64 age group. Females exhibited a significantly higher likelihood, being 1.78 times more likely (95% CI: 1.54–2.06) to report chronic diseases compared to males. When considering population groups, Indian and Asian older individuals were 0.37 times less likely (95% CI: 0.24–0.56) to report chronic diseases compared to the Black population group. Similarly, Coloured individuals were 0.51 times less likely (95% CI: 0.38–0.67), and White individuals were 0.39 times less likely (95% CI: 0.30–0.49) to report chronic diseases compared to the Black population group.

**TABLE 3:** Binary logistic regression on the determinants of self-reported chronic disease diagnoses.

Characteristics	AOR	s.d.	*t*	95% CI	
				Lower	Upper
**Age group**					
60–64†	1.00	-	-	-	-
65–69	1.11	0.08	1.43	0.96	1.28
70–74	1.65***	0.15	5.64	1.39	1.97
75–79	1.37**	0.15	2.87	1.11	1.70
80+	1.40**	0.16	2.95	1.12	1.75
**Sex**					
Male†	1.00	-	-	-	-
Female	1.78***	0.13	7.65	1.54	2.06
**Population group**					
Black people†	1.00	-	-	-	-
Coloured people	0.51***	0.07	-4.81	0.38	0.67
Indian people and Asian people	0.37***	0.08	-4.68	0.24	0.56
White people	0.39***	0.05	-7.80	0.30	0.49
**Marital status**					
Married	1.39*	0.22	2.10	1.02	1.90
Cohabiting†	1.00	-	-	-	-
Never married	1.28	0.22	1.43	0.91	1.81
No longer married	1.75***	0.29	3.42	1.27	2.42
**Educational level**					
No education†	1.00	-	-	-	-
Primary	1.19*	0.10	2.01	1.00	1.40
Secondary	1.19	0.11	1.76	0.98	1.43
Higher	1.04	0.15	0.30	0.79	1.38
Other	1.13	0.22	0.63	0.77	1.65
**Disability status**					
No difficulty†	1.00	-	-	-	-
Some difficulty	1.42***	0.10	4.92	1.23	1.63
A lot of difficulty	1.32**	0.13	2.90	1.09	1.59
Cannot do at all	1.31	0.23	1.51	0.92	1.86
**Household wealth status**					
Poor	0.73**	0.07	-3.13	0.59	0.89
Average	0.83*	0.08	-2.01	0.69	1.00
Rich†	1.00	-	-	-	-
**Household composition**					
Lone male	0.78	0.12	-1.59	0.57	1.06
Lone female	0.52***	0.09	-3.89	0.38	0.73
Nuclear, male-headed†	1.00	-	-	-	-
Nuclear, female-headed	0.61**	0.10	-3.16	0.45	0.83
Extended, male-headed	0.70***	0.07	-3.58	0.58	0.85
Extended, female-headed	0.69**	0.09	-2.79	0.53	0.89
Complex	0.64	0.15	-1.95	0.41	1.00
**Geography type**					
Urban†	1.00	-	-	-	-
Traditional	0.96	0.09	-0.44	0.80	1.15
Farms	1.20	0.23	0.97	0.83	1.75
**Province**					
Western Cape	2.01***	0.34	4.19	1.45	2.80
Eastern Cape	2.24***	0.25	7.13	1.80	2.80
Northern Cape	1.86***	0.34	3.37	1.30	2.67
Free State	1.82***	0.33	3.32	1.28	2.59
KwaZulu-Natal	2.68***	0.32	8.14	2.11	3.39
North West	1.98***	0.27	4.97	1.51	2.60
Gauteng	1.93***	0.25	5.00	1.49	2.50
Mpumalanga	1.92***	0.27	4.69	1.46	2.52
Limpopo†	1.00	-	-	-	-
_cons	0.32	0.07	-5.23	0.21	0.49

AOR, adjusted odds ratio; s.d., standard error; CI, confidence interval.

† , reference category

* , *p* < 0.05;

** , *p* < 0.01;

*** , *p* < 0.001.

The study revealed that older individuals who were previously married were 1.75 times more likely (95% CI: 1.27–2.42) to report self-reported chronic diseases compared to those who were cohabiting. Similarly, those currently married exhibited a 1.39 times higher likelihood (95% CI: 1.02–1.90) of reporting chronic diseases compared to their cohabiting counterparts. Individuals with primary education attainment showed a 1.19 times higher likelihood (95% CI: 1.00–1.40) of reporting chronic diseases compared to those with no education. Those categorised as having ‘some difficulty’ in terms of disability status were 1.42 times more likely (95% CI: 1.23–1.63) to report chronic diseases compared to those reporting ‘no difficulty’ in disability. Similarly, older individuals classified as experiencing ‘a lot of difficulty’ in disability status were 1.32 times more likely (95% CI: 1.09–1.59) to report chronic diseases compared to those with ‘no difficulty’ in disability. Individuals from poor households had a 0.73 times lower likelihood (95% CI: 0.59–0.89) of reporting self-reported chronic diseases compared to those from rich households.

Likewise, individuals from households with average wealth exhibited a 0.83 times reduced likelihood [95% CI: 0.69–1.00] of being diagnosed with self-reported chronic diseases than those from rich households. In summary, the likelihood of being diagnosed with chronic conditions decreased as wealth status decreased. The results indicate that the likelihood of being diagnosed with self-reported chronic diseases was significantly predicted by household composition. The older individuals living in lone households that were female-headed exhibited a 0.52 times lower likelihood [95% CI: 0.38–0.73] of being diagnosed with self-reported chronic diseases compared to their counterparts in male-headed nuclear households.

Similarly, individuals of older age residing in male-headed extended households showed a 0.70 times decreased likelihood [95% CI: 0.58–0.85] of receiving diagnoses for self-reported chronic diseases than those residing in households that were nuclear male-headed. In households that were nuclear female headed, there was a 0.61 times reduced likelihood [95% CI: 0.45–0.83] of being diagnosed with self-reported chronic diseases compared to households that were nuclear male-headed. Additionally, individuals from households that were extended female-headed exhibited a 0.69 times lower likelihood [95% CI: 0.53–0.89] of being diagnosed with self-reported chronic diseases than those from households that were nuclear male-headed.

Older individuals who resided in the Western Cape had a 2.01 times higher likelihood [95% CI: 1.45–2.80] of being diagnosed with self-reported chronic diseases than older individuals from Limpopo. Similarly, those who resided in the Eastern Cape had a 2.24 times higher likelihood [95% CI: 1.80–2.80] of being diagnosed with self-reported chronic diseases than older individuals from Limpopo. Older persons residing in Gauteng had a 1.93 times higher likelihood [95% CI: 1.49–2.50] of being diagnosed with self-reported chronic diseases than older individuals from Limpopo. Individuals of older age residing in KwaZulu-Natal showed a 2.68 times increased likelihood [95% CI: 2.11–3.39] of being diagnosed with self-reported chronic diseases compared to those from the Limpopo province.

## Discussion

This study sought to examine the determinants of self-reported chronic disease diagnoses among older individuals residing in South Africa. We found that age, population group, sex, marital status, level of education, disability status, household composition and province had an association with self-reported chronic disease diagnoses among older persons in South Africa. These factors have been found to have an association with self-reported chronic condition diagnoses in previous studies.^31,32,33,34^ We found that at least 5 in 10 older persons reported being diagnosed with chronic diseases. Persons aged 70 years and older had higher odds of self-reported chronic disease diagnoses. Similar studies show that those in their middle older years tend to have higher odds of having chronic diseases.^17,35,36^ This finding suggests that as age increases, health deteriorates and one becomes more susceptible to a variety of chronic conditions. As individuals grow older, there is typically an increased risk of developing chronic diseases and experiencing a decline in overall health. We also found higher odds of self-reported chronic disease diagnoses among older females compared to males. Previous research has found that females exhibit a higher prevalence (and odds) of chronic diseases compared to males.^37,38^ Although most chronic conditions are not gender-specific, females tend to be affected by chronic diseases at a higher rate than males.^39^ Studies have highlighted a lack of physical activity and obesity as primary factors contributing to the higher prevalence of self-reported chronic disease diagnoses among females compared to males.^17,40^

We also found racial differences in self-reported chronic disease diagnoses; we found that, compared to the black population group, those from the non-black population groups had lower odds of self-reported chronic disease diagnoses. Similar studies have also found racial differences in chronic conditions.^41^ South Africa has racial and socioeconomic disparities in access to better healthcare.^42^ Many of those from the black population group live in poor socioeconomic conditions and this could be a factor in their poor health outcomes. Research suggests that the economic status of black individuals contributes to their poorer health status.^43^ Moreover, we found that, compared to older persons who were cohabiting, those who were married as well as those who were no longer married had higher odds of being diagnosed with self-reported chronic diseases. Studies show that the prevalence of chronic diseases in older unmarried persons is higher than in older married persons.^18,44,45^ Several studies show that married people may have better health results for a variety of reasons, and married older individuals tend to experience better health outcomes compared to their unmarried counterparts.^18,46,47^ As a result of marital selection, healthier people may be more likely to marry and stay married for longer, whereas less healthy people may be more likely to be single, separated, or divorced.^48,49^ Conversely, the concept of the marital protection effect suggests that married individuals tend to benefit from various advantages including greater access to economic resources, social and psychological support and healthier behaviours. Additionally, divorce is recognised as a significant source of stress that can adversely affect one’s health.^35,50^

Moreover, we found that the odds of being diagnosed with chronic diseases were higher among those with primary education compared to those with no education. Low educational attainment is a risk factor for chronic conditions in several studies.^51,52^ Those with lower educational levels tend to have poor socioeconomic status and lack the economic resources to take better care of their health. Higher educational attainment may mean better knowledge of various chronic conditions and how one needs to take better care of oneself, and this may not always be the case among those with lower levels of education. We also found a relationship between disability status and being diagnosed with chronic diseases. Our study revealed that among older individuals, those with ‘some difficulty’ and those with ‘a lot of difficulty’ had higher odds of being diagnosed with self-reported chronic diseases. There is a significant difference between people with disabilities who have all chronic conditions and people without disabilities.^53^ Older individuals with disabilities face an increased risk of being diagnosed with self-reported chronic diseases.^53,54^ We further found that the odds of being diagnosed with self-reported chronic diseases increased with household wealth status, whereby those from poor and average-wealth households had lower odds of being diagnosed with self-reported chronic diseases. This finding is in line with findings from similar studies.^55,56^ This could be that households with better wealth status tend to also have higher levels of obesity and sedentary lifestyles which could increase the likelihood of being diagnosed with self-reported chronic diseases.

We also found that household living arrangements (household composition) were another key determinant of being diagnosed with self-reported chronic diseases. We found that older persons from lone female households, female-headed nuclear households and extended households (both male- and female-headed) had lower odds of self-reported chronic disease diagnoses than those from male-headed nuclear households. Some studies have revealed that there is a relationship between household living arrangements and chronic conditions, where households consisting of one or two individuals tend to report more chronic diseases compared to larger households.^57,58^ People who live alone are more prone to adopting unhealthy lifestyles, which can have adverse effects on their health. Household members play a role in promoting healthy behaviours by acting as social controls; with fewer individuals living in the household, there can be better interventions for unhealthy behaviours in the home, which can negatively affect health.^59,60,61^ Furthermore, while we found that those from most of the provinces had significantly higher odds of chronic disease diagnoses than those from Limpopo, the odds were almost three-fold among those from KwaZulu-Natal. There are a few potential explanations for the higher odds of chronic disease diagnoses, particularly in KwaZulu-Natal. The province generally has higher levels of economic development compared to Limpopo. Higher socioeconomic status is associated with better access to healthcare, healthier lifestyle choices and increased awareness of chronic diseases. Moreover, KwaZulu-Natal is among the provinces with a large population size^26^; a larger population size tends to lead to an increased likelihood of chronic diseases. Research has also shown that KwaZulu-Natal is among those with a higher number of deaths because of NCDs, which could also be attributed to the population size in the province.^62^

### Strengths and limitations

It is widely acknowledged in research that a certain degree of bias may be unavoidable in studies. The GHS (2019) excluded the population living in institutions (hospitals, old-age homes, etc.) from the sample. Excluding older individuals living in institutions means that the results may not fully represent this significant segment of the older population. Consequently, from this dataset, it is challenging to ascertain the living arrangements of this population, including those residing in old-age homes or hospitals. The GHS also did not include information on lifestyle-related factors (i.e., food the respondents eat, whether they exercise or not, whether they smoke or not, whether they drink alcohol or not, etc.). Access to health facilities could also be a limitation, in that some provinces have healthcare services that are closer to the people. This is not the case in predominantly rural provinces, where people have to travel long distances to access healthcare facilities. However, because of the methodology used to analyse this data, the findings of this study are generalisable to the population of persons aged 60 years and older in South Africa.

## Conclusion

The study’s findings revealed significant statistical associations between the diagnoses of chronic diseases among the older population in South Africa and various demographic factors, including age, sex, marital status, educational level, disability status, household composition and the province of residence. The findings also revealed a higher prevalence of self-reported chronic disease diagnoses among older females compared to males. These research insights offer valuable information regarding the relationships between socio-demographic factors and chronic diseases among the elderly. The implications of the study findings extend to the health system, policymakers and all stakeholders involved in the sector. To gain a deeper understanding of chronic conditions among older individuals, we recommend the inclusion of lifestyle factor-related questions in future GHSs. By incorporating such questions, more information can be gathered about the lifestyle choices and behaviours that may contribute to the development or prevention of chronic diseases in this vulnerable population. Besides the inclusion of lifestyle-related questions in the GHS, there is a need for the implementation of longitudinal studies to explore the impact of socio-demographic factors such as education and age on the development and progression of chronic diseases among older persons.
